# Methyl 2-((2*Z*,5*Z*)-4-oxo-3-phenyl-2-{2-[(1*E*)-1,2,3,4-tetra­hydro­naphthalen-1-yl­idene]hydrazin-1-yl­idene}-1,3-thia­zolidin-5-yl­idene)acetate

**DOI:** 10.1107/S1600536814005285

**Published:** 2014-03-15

**Authors:** Joel T. Mague, Mehmet Akkurt, Shaaban K. Mohamed, Alaa A. Hassan, Mustafa R. Albayati

**Affiliations:** a Department of Chemistry, Tulane University, New Orleans, LA 70118, USA; b Department of Physics, Faculty of Sciences, Erciyes University, 38039 Kayseri, Turkey; c Chemistry and Environmental Division, Manchester Metropolitan University, Manchester M1 5GD, England; d Chemistry Department, Faculty of Science, Mini University, 61519 El-Minia, Egypt; e Kirkuk University, College of Science, Department of Chemistry, Kirkuk, Iraq

## Abstract

In the title compound, C_22_H_19_N_3_O_3_S, the six-membered ring of the 1,2,3,4-tetra­hydro­naphthalene ring system adopts an envelope conformation with the central CH_2_ C atom as the flap. The mol­ecular conformation is stabilized by an S⋯O contact, forming a pseudo-five-membered ring. In the crystal, mol­ecules are linked *via* C—H⋯O hydrogen bonds into chains propagating along [102].

## Related literature   

For the synthesis of thia­zolidinediones, see: Patel *et al.* (2010 ▶); Aneja *et al.* (2011 ▶). For pharmacological properties of thia­zolidinedione-containing compounds, see: Gillies & Dunn (2000 ▶); Lenhard & Funk (2001 ▶); Edelman (2003 ▶); Desmet *et al.* (2005 ▶). For ring conformation, see: Cremer & Pople (1975 ▶). For the synthesis of the title compound, see: Mague *et al.* (2014 ▶).
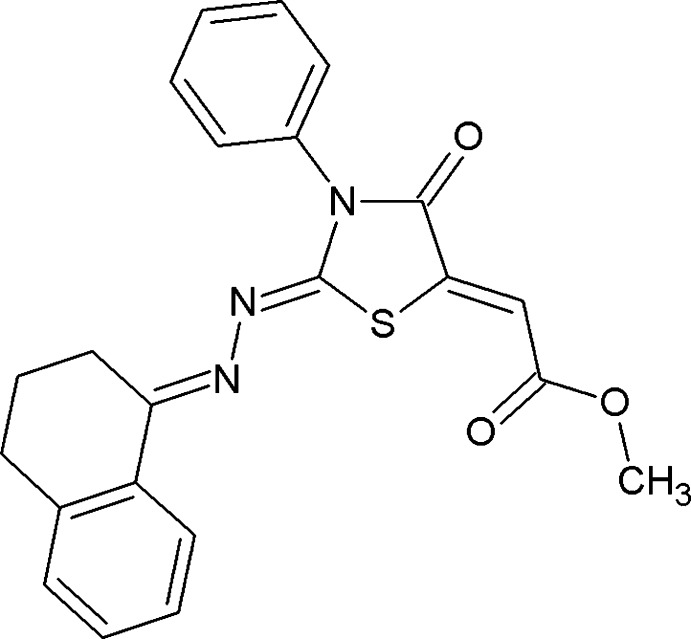


## Experimental   

### 

#### Crystal data  

C_22_H_19_N_3_O_3_S
*M* *_r_* = 405.47
Triclinic, 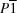

*a* = 9.7078 (6) Å
*b* = 9.7134 (6) Å
*c* = 11.1061 (7) Å
α = 67.9810 (9)°
β = 88.8400 (9)°
γ = 85.9840 (9)°
*V* = 968.47 (10) Å^3^
*Z* = 2
Mo *K*α radiation
μ = 0.20 mm^−1^
*T* = 150 K
0.28 × 0.16 × 0.09 mm

#### Data collection  

Bruker SMART APEX CCD diffractometer
Absorption correction: multi-scan (*SADABS*; Bruker, 2013 ▶) *T*_min_ = 0.83, *T*_max_ = 0.98
17735 measured reflections
4994 independent reflections
4325 reflections with *I* > 2σ(*I*)
*R*_int_ = 0.036

#### Refinement  

*R*[*F* ^2^ > 2σ(*F* ^2^)] = 0.040
*wR*(*F* ^2^) = 0.104
*S* = 1.05
4994 reflections
263 parameters
H-atom parameters constrained
Δρ_max_ = 0.39 e Å^−3^
Δρ_min_ = −0.30 e Å^−3^

### 

Data collection: *APEX2* (Bruker, 2013 ▶); cell refinement: *SAINT* (Bruker, 2013 ▶); data reduction: *SAINT*; program(s) used to solve structure: *SHELXS97* (Sheldrick, 2008 ▶); program(s) used to refine structure: *SHELXL2013* (Sheldrick, 2008 ▶); molecular graphics: *ORTEP-3 for Windows* (Farrugia, 2012 ▶); software used to prepare material for publication: *WinGX* (Farrugia, 2012 ▶) and *PLATON* (Spek, 2009 ▶).

## Supplementary Material

Crystal structure: contains datablock(s) global, I. DOI: 10.1107/S1600536814005285/bt6966sup1.cif

Structure factors: contains datablock(s) I. DOI: 10.1107/S1600536814005285/bt6966Isup2.hkl

Supporting information file. DOI: 10.1107/S1600536814005285/bt6966Isup3.cml

CCDC reference: 990619

Additional supporting information:  crystallographic information; 3D view; checkCIF report

## Figures and Tables

**Table 1:** Hydrogen-bond geometry (Å, °)

*D*—H⋯*A*	*D*—H	H⋯*A*	*D*⋯*A*	*D*—H⋯*A*
C5—H5⋯O2^i^	0.95	2.59	3.3704 (18)	140
C11—H11*B*⋯N2	0.99	2.38	2.7466 (18)	101
C20—H20⋯O3^ii^	0.95	2.56	3.4591 (16)	159
C22—H22*C*⋯O1^ii^	0.98	2.52	3.4397 (18)	157

Symmetry codes: (i) 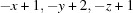; (ii) 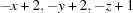.

**Fig. 1.**
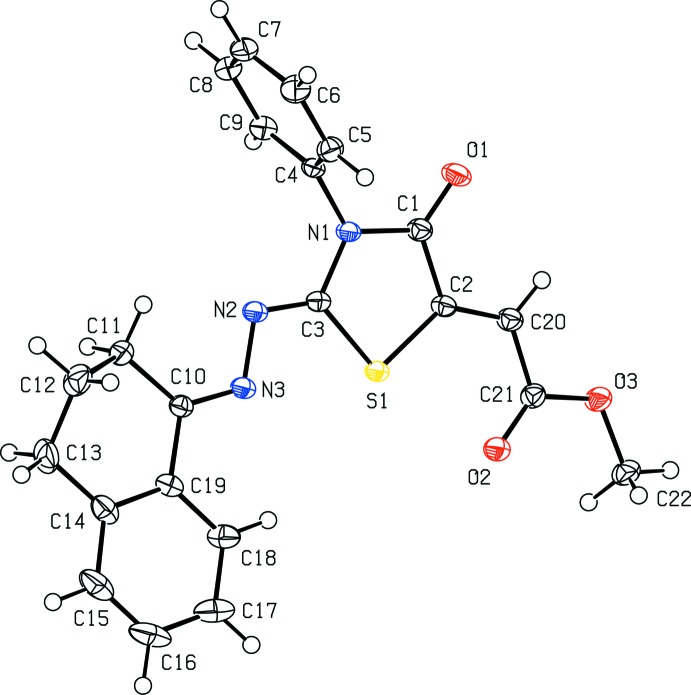
Perspective view of the title molecule with 50% probability ellipsoids.

**Fig. 2.**
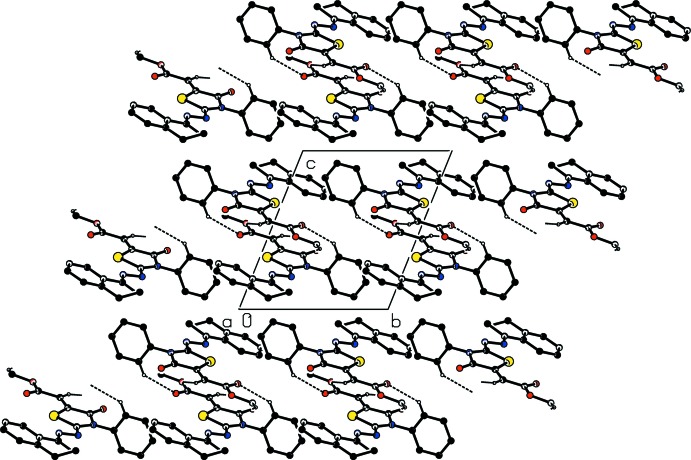
Packing and hydrogen-bonding interactions of the title compound viewed down the a axis. H atoms not involved in H bonding are omitted for clarity.

## supplementary crystallographic information

### 1. Comment

Thiazolidinediones (TZDs) are a pharmacological group of structurally related compounds characterized by a thiazolidinedione ring to which divergent molecular moieties are attached. Many methods have been reoprted for the synthesis of thiazolidinedione-containing compounds and their analogues (Patel *et al.*, 2010; Aneja *et al.*, 2011). The most beneﬁcial effects of TZDs such as rosiglitazone and pioglitazone is the treatment of type II diabetes by lowering blood glucose levels *via* increasing insulin sensitivity with no hepatic side effects (Gillies & Dunn, 2000; Lenhard & Funk, 2001). They were shown also to reduce cardiovascular risk factors associated with this condition (Edelman, 2003). Recently, an anti-inﬂammatory potential for TZDs has also been suggested (Desmet *et al.*, 2005). In this context we synthesized the title compound as part of our on-going study in synthesis and biological reactivities of thiazolidinedione scaffold compounds.

In the title compound, (Fig. 1), a puckering analysis (Cremer & Pople, 1975) of the six-membered ring C10/C11/C12/C13/C14/C19 indicates it to adopt the "envelope" conformation with puckering parameters Q_T_ = 0.4855 (17) Å, θ = 126.50 (19)° and φ = 296.6 (2)°. The 1,3-thiazolidine ring (S1/N1/C1—C3) make dihedral angles of 12.22 (7) and 85.72 (6) °, respectively, with the benzene ring (C14–C19) of the 1,2,3,4-tetrahydronaphthalene ring system and the phenyl ring (C4–C9). The C3–N2–N3–C10, C2–C20–C21–O3 and C20–C21–O3–C22 torsion angles are 179.44 (11), 176.24 (12) and -177.15 (11) °, respectively.

The molecular conformation of the title compound is stabilized by a S1···O2 contact forming a pseudo five-membered ring. In the crystal packing, C—H···O hydrogen bonds connect the molecules generating chains running along [1 0 2] (Table 1, Fig. 2).

### 2. Experimental

The title compound was synthesized based on our previous reported method (Mague *et al.*, 2014). Orange crystals (m.p.501–503 K) suitable for X-ray analysis were grown from an ethanolic solution at room temperature after two days.

### 3. Refinement

All H atoms were placed in geometrically idealized positions and constrained to ride on their parent atoms with C—H = 0.95–0.99 Å, with *U*_iso_(H) = 1.2 or 1.5 *U*_iso_(C).

### Crystal data

**Table:**

C_22_H_19_N_3_O_3_S	*Z* = 2
*M**_r_* = 405.47	*F*(000) = 424
Triclinic, *P*1	*D*_x_ = 1.390 Mg m^−^^3^
Hall symbol: -P 1	Mo *K*α radiation, λ = 0.71073 Å
*a* = 9.7078 (6) Å	Cell parameters from 9980 reflections
*b* = 9.7134 (6) Å	θ = 2.3–29.2°
*c* = 11.1061 (7) Å	µ = 0.20 mm^−^^1^
α = 67.9810 (9)°	*T* = 150 K
β = 88.8400 (9)°	Thick plate, orange
γ = 85.9840 (9)°	0.28 × 0.16 × 0.09 mm
*V* = 968.47 (10) Å^3^	

### Data collection

**Table:**

Bruker SMART APEX CCD diffractometer	4994 independent reflections
Radiation source: fine-focus sealed tube	4325 reflections with *I* > 2σ(*I*)
Graphite monochromator	*R*_int_ = 0.036
Detector resolution: 8.3660 pixels mm^-1^	θ_max_ = 29.2°, θ_min_ = 2.0°
φ and ω scans	*h* = −13→13
Absorption correction: multi-scan (*SADABS*; Bruker, 2013)	*k* = −13→13
*T*_min_ = 0.83, *T*_max_ = 0.98	*l* = −14→15
17735 measured reflections	

### Refinement

**Table:**

Refinement on *F*^2^	0 restraints
Least-squares matrix: full	Hydrogen site location: inferred from neighbouring sites
*R*[*F*^2^ > 2σ(*F*^2^)] = 0.040	H-atom parameters constrained
*wR*(*F*^2^) = 0.104	*w* = 1/[σ^2^(*F*_o_^2^) + (0.0495*P*)^2^ + 0.3384*P*] where *P* = (*F*_o_^2^ + 2*F*_c_^2^)/3
*S* = 1.05	(Δ/σ)_max_ = 0.001
4994 reflections	Δρ_max_ = 0.39 e Å^−^^3^
263 parameters	Δρ_min_ = −0.30 e Å^−^^3^

### Special details

**Table:**

Geometry. Bond distances, angles *etc*. have been calculated using the rounded fractional coordinates. All su's are estimated from the variances of the (full) variance-covariance matrix. The cell e.s.d.'s are taken into account in the estimation of distances, angles and torsion angles
Refinement. Refinement on *F*^2^ for ALL reflections except those flagged by the user for potential systematic errors. Weighted *R*-factors *wR* and all goodnesses of fit *S* are based on *F*^2^, conventional *R*-factors *R* are based on *F*, with *F* set to zero for negative *F*^2^. The observed criterion of *F*^2^ > σ(*F*^2^) is used only for calculating -*R*-factor-obs *etc*. and is not relevant to the choice of reflections for refinement. *R*-factors based on *F*^2^ are statistically about twice as large as those based on *F*, and *R*-factors based on ALL data will be even larger.

### Fractional atomic coordinates and isotropic or equivalent isotropic displacement parameters (Å^2^)

**Table:**

	*x*	*y*	*z*	*U*_iso_*/*U*_eq_	
S1	0.52540 (3)	0.95022 (3)	0.67094 (3)	0.0188 (1)	
O1	0.76785 (10)	0.63932 (11)	0.62875 (11)	0.0310 (3)	
O2	0.66788 (9)	1.19374 (10)	0.53017 (10)	0.0249 (3)	
O3	0.88571 (9)	1.18392 (10)	0.45791 (10)	0.0255 (3)	
N1	0.56204 (10)	0.66168 (12)	0.72779 (11)	0.0189 (3)	
N2	0.34944 (11)	0.73712 (12)	0.79413 (11)	0.0200 (3)	
N3	0.27229 (11)	0.87024 (12)	0.78071 (11)	0.0209 (3)	
C1	0.67901 (13)	0.71539 (14)	0.65725 (13)	0.0208 (3)	
C2	0.67731 (12)	0.88043 (14)	0.62092 (13)	0.0192 (3)	
C3	0.46688 (12)	0.76959 (14)	0.74001 (12)	0.0178 (3)	
C4	0.52709 (12)	0.50951 (14)	0.76333 (13)	0.0185 (3)	
C5	0.46558 (14)	0.47045 (15)	0.67052 (13)	0.0237 (3)	
C6	0.42866 (15)	0.32512 (16)	0.70419 (15)	0.0286 (4)	
C7	0.45075 (15)	0.22246 (15)	0.82939 (15)	0.0274 (4)	
C8	0.51370 (15)	0.26328 (15)	0.92091 (14)	0.0267 (4)	
C9	0.55316 (14)	0.40784 (15)	0.88794 (13)	0.0234 (4)	
C10	0.15034 (12)	0.85321 (14)	0.83051 (12)	0.0188 (3)	
C11	0.09413 (14)	0.70582 (15)	0.90877 (14)	0.0251 (4)	
C12	−0.06198 (15)	0.71071 (17)	0.89171 (16)	0.0333 (5)	
C13	−0.13270 (15)	0.83434 (19)	0.92826 (16)	0.0363 (5)	
C14	−0.07537 (14)	0.98191 (17)	0.85397 (14)	0.0271 (4)	
C15	−0.15709 (16)	1.1146 (2)	0.82824 (16)	0.0369 (5)	
C16	−0.10746 (18)	1.25107 (19)	0.75859 (17)	0.0400 (5)	
C17	0.02647 (18)	1.25928 (18)	0.71144 (17)	0.0379 (5)	
C18	0.10916 (15)	1.12992 (16)	0.73521 (15)	0.0289 (4)	
C19	0.06062 (13)	0.99048 (15)	0.80716 (13)	0.0216 (3)	
C20	0.78083 (13)	0.96017 (14)	0.55569 (13)	0.0219 (4)	
C21	0.77041 (13)	1.12321 (14)	0.51469 (13)	0.0210 (3)	
C22	0.88037 (15)	1.34487 (15)	0.40941 (15)	0.0282 (4)	
H5	0.44880	0.54180	0.58520	0.0280*	
H6	0.38810	0.29610	0.64100	0.0340*	
H7	0.42280	0.12400	0.85270	0.0330*	
H8	0.52990	0.19220	1.00640	0.0320*	
H9	0.59720	0.43610	0.94990	0.0280*	
H11A	0.11620	0.67920	1.00170	0.0300*	
H11B	0.13900	0.62790	0.88110	0.0300*	
H12A	−0.08400	0.72830	0.80030	0.0400*	
H12B	−0.09670	0.61390	0.94740	0.0400*	
H13A	−0.23290	0.84120	0.91030	0.0440*	
H13B	−0.12030	0.80970	1.02250	0.0440*	
H15	−0.24910	1.11020	0.85960	0.0440*	
H16	−0.16480	1.33950	0.74280	0.0480*	
H17	0.06110	1.35320	0.66310	0.0450*	
H18	0.20050	1.13580	0.70210	0.0350*	
H20	0.86120	0.91130	0.53580	0.0260*	
H22A	0.84870	1.37910	0.47840	0.0420*	
H22B	0.81610	1.38580	0.33560	0.0420*	
H22C	0.97260	1.37860	0.38140	0.0420*	

### Atomic displacement parameters (Å^2^)

**Table:**

	*U*^11^	*U*^22^	*U*^33^	*U*^12^	*U*^13^	*U*^23^
S1	0.0177 (2)	0.0149 (2)	0.0232 (2)	0.0008 (1)	0.0016 (1)	−0.0071 (1)
O1	0.0221 (5)	0.0214 (5)	0.0509 (7)	−0.0001 (4)	0.0105 (4)	−0.0159 (5)
O2	0.0224 (4)	0.0198 (5)	0.0310 (5)	0.0003 (4)	0.0032 (4)	−0.0081 (4)
O3	0.0204 (4)	0.0180 (5)	0.0359 (6)	−0.0037 (4)	0.0039 (4)	−0.0075 (4)
N1	0.0172 (5)	0.0147 (5)	0.0251 (5)	0.0007 (4)	0.0011 (4)	−0.0082 (4)
N2	0.0197 (5)	0.0161 (5)	0.0242 (6)	0.0011 (4)	0.0011 (4)	−0.0080 (4)
N3	0.0198 (5)	0.0170 (5)	0.0258 (6)	0.0012 (4)	0.0021 (4)	−0.0086 (4)
C1	0.0174 (5)	0.0184 (6)	0.0269 (7)	−0.0008 (5)	0.0006 (5)	−0.0090 (5)
C2	0.0173 (5)	0.0175 (6)	0.0232 (6)	0.0013 (4)	−0.0009 (4)	−0.0085 (5)
C3	0.0190 (5)	0.0153 (5)	0.0189 (6)	0.0019 (4)	−0.0021 (4)	−0.0067 (5)
C4	0.0158 (5)	0.0145 (6)	0.0255 (6)	0.0007 (4)	0.0032 (4)	−0.0082 (5)
C5	0.0269 (6)	0.0208 (6)	0.0230 (6)	−0.0017 (5)	0.0002 (5)	−0.0079 (5)
C6	0.0331 (7)	0.0254 (7)	0.0317 (8)	−0.0063 (6)	0.0014 (6)	−0.0150 (6)
C7	0.0312 (7)	0.0177 (6)	0.0351 (8)	−0.0037 (5)	0.0070 (6)	−0.0118 (6)
C8	0.0310 (7)	0.0187 (6)	0.0268 (7)	0.0020 (5)	0.0032 (5)	−0.0051 (5)
C9	0.0238 (6)	0.0210 (6)	0.0255 (7)	0.0015 (5)	−0.0009 (5)	−0.0093 (5)
C10	0.0196 (6)	0.0175 (6)	0.0202 (6)	−0.0001 (5)	0.0006 (4)	−0.0084 (5)
C11	0.0232 (6)	0.0197 (6)	0.0282 (7)	−0.0013 (5)	0.0050 (5)	−0.0046 (5)
C12	0.0234 (7)	0.0295 (8)	0.0400 (9)	−0.0071 (6)	0.0032 (6)	−0.0042 (6)
C13	0.0218 (7)	0.0440 (9)	0.0346 (8)	0.0017 (6)	0.0088 (6)	−0.0061 (7)
C14	0.0227 (6)	0.0355 (8)	0.0237 (7)	0.0064 (6)	−0.0008 (5)	−0.0132 (6)
C15	0.0289 (7)	0.0483 (10)	0.0359 (8)	0.0159 (7)	−0.0031 (6)	−0.0216 (8)
C16	0.0446 (9)	0.0360 (9)	0.0442 (10)	0.0217 (7)	−0.0139 (7)	−0.0239 (8)
C17	0.0476 (9)	0.0214 (7)	0.0460 (10)	0.0071 (7)	−0.0107 (7)	−0.0153 (7)
C18	0.0293 (7)	0.0211 (7)	0.0382 (8)	0.0016 (5)	−0.0029 (6)	−0.0137 (6)
C19	0.0213 (6)	0.0216 (6)	0.0243 (6)	0.0035 (5)	−0.0020 (5)	−0.0120 (5)
C20	0.0185 (6)	0.0193 (6)	0.0276 (7)	0.0005 (5)	0.0003 (5)	−0.0088 (5)
C21	0.0202 (6)	0.0193 (6)	0.0226 (6)	−0.0026 (5)	−0.0001 (5)	−0.0066 (5)
C22	0.0257 (6)	0.0179 (6)	0.0379 (8)	−0.0050 (5)	0.0038 (6)	−0.0066 (6)

### Geometric parameters (Å, º)

**Table:**

S1—C2	1.7470 (13)	C14—C19	1.4054 (19)
S1—C3	1.7614 (14)	C15—C16	1.376 (3)
O1—C1	1.2104 (17)	C16—C17	1.388 (2)
O2—C21	1.2147 (16)	C17—C18	1.383 (2)
O3—C21	1.3354 (16)	C18—C19	1.399 (2)
O3—C22	1.4475 (19)	C20—C21	1.471 (2)
N1—C1	1.3841 (17)	C5—H5	0.9500
N1—C3	1.3916 (18)	C6—H6	0.9500
N1—C4	1.4433 (19)	C7—H7	0.9500
N2—N3	1.4069 (17)	C8—H8	0.9500
N2—C3	1.2810 (16)	C9—H9	0.9500
N3—C10	1.2912 (16)	C11—H11A	0.9900
C1—C2	1.498 (2)	C11—H11B	0.9900
C2—C20	1.3392 (18)	C12—H12A	0.9900
C4—C5	1.383 (2)	C12—H12B	0.9900
C4—C9	1.3817 (19)	C13—H13A	0.9900
C5—C6	1.390 (2)	C13—H13B	0.9900
C6—C7	1.384 (2)	C15—H15	0.9500
C7—C8	1.388 (2)	C16—H16	0.9500
C8—C9	1.392 (2)	C17—H17	0.9500
C10—C11	1.503 (2)	C18—H18	0.9500
C10—C19	1.480 (2)	C20—H20	0.9500
C11—C12	1.526 (2)	C22—H22A	0.9800
C12—C13	1.522 (3)	C22—H22B	0.9800
C13—C14	1.501 (2)	C22—H22C	0.9800
C14—C15	1.401 (3)		
			
C2—S1—C3	90.54 (6)	O3—C21—C20	112.24 (11)
C21—O3—C22	115.22 (11)	C4—C5—H5	121.00
C1—N1—C3	115.28 (12)	C6—C5—H5	121.00
C1—N1—C4	122.38 (11)	C5—C6—H6	120.00
C3—N1—C4	121.19 (10)	C7—C6—H6	120.00
N3—N2—C3	108.54 (11)	C6—C7—H7	120.00
N2—N3—C10	114.88 (12)	C8—C7—H7	120.00
O1—C1—N1	124.49 (13)	C7—C8—H8	120.00
O1—C1—C2	125.72 (12)	C9—C8—H8	120.00
N1—C1—C2	109.78 (11)	C4—C9—H9	121.00
S1—C2—C1	111.61 (9)	C8—C9—H9	121.00
S1—C2—C20	126.19 (12)	C10—C11—H11A	109.00
C1—C2—C20	122.19 (12)	C10—C11—H11B	109.00
S1—C3—N1	112.60 (9)	C12—C11—H11A	109.00
S1—C3—N2	125.31 (11)	C12—C11—H11B	109.00
N1—C3—N2	122.08 (13)	H11A—C11—H11B	108.00
N1—C4—C5	118.25 (12)	C11—C12—H12A	110.00
N1—C4—C9	119.99 (12)	C11—C12—H12B	110.00
C5—C4—C9	121.75 (13)	C13—C12—H12A	110.00
C4—C5—C6	118.88 (13)	C13—C12—H12B	110.00
C5—C6—C7	120.30 (14)	H12A—C12—H12B	108.00
C6—C7—C8	120.01 (14)	C12—C13—H13A	109.00
C7—C8—C9	120.27 (13)	C12—C13—H13B	109.00
C4—C9—C8	118.76 (13)	C14—C13—H13A	109.00
N3—C10—C11	124.91 (13)	C14—C13—H13B	109.00
N3—C10—C19	116.60 (12)	H13A—C13—H13B	108.00
C11—C10—C19	118.49 (11)	C14—C15—H15	119.00
C10—C11—C12	111.39 (12)	C16—C15—H15	119.00
C11—C12—C13	110.22 (13)	C15—C16—H16	120.00
C12—C13—C14	111.71 (13)	C17—C16—H16	120.00
C13—C14—C15	120.61 (13)	C16—C17—H17	120.00
C13—C14—C19	120.99 (14)	C18—C17—H17	120.00
C15—C14—C19	118.40 (15)	C17—C18—H18	119.00
C14—C15—C16	121.64 (15)	C19—C18—H18	119.00
C15—C16—C17	119.89 (17)	C2—C20—H20	120.00
C16—C17—C18	119.62 (17)	C21—C20—H20	120.00
C17—C18—C19	121.12 (14)	O3—C22—H22A	109.00
C10—C19—C14	120.36 (13)	O3—C22—H22B	109.00
C10—C19—C18	120.29 (12)	O3—C22—H22C	109.00
C14—C19—C18	119.32 (14)	H22A—C22—H22B	109.00
C2—C20—C21	120.19 (12)	H22A—C22—H22C	109.00
O2—C21—O3	124.27 (13)	H22B—C22—H22C	110.00
O2—C21—C20	123.49 (12)		
			
C3—S1—C2—C1	1.03 (10)	C9—C4—C5—C6	0.3 (2)
C3—S1—C2—C20	179.69 (13)	N1—C4—C9—C8	177.63 (12)
C2—S1—C3—N1	1.64 (10)	C5—C4—C9—C8	−1.3 (2)
C2—S1—C3—N2	−177.12 (12)	C4—C5—C6—C7	1.3 (2)
C22—O3—C21—O2	−2.31 (19)	C5—C6—C7—C8	−1.9 (2)
C22—O3—C21—C20	177.15 (11)	C6—C7—C8—C9	0.9 (2)
C3—N1—C1—O1	−174.48 (13)	C7—C8—C9—C4	0.7 (2)
C3—N1—C1—C2	4.83 (15)	N3—C10—C11—C12	−149.23 (14)
C4—N1—C1—O1	−6.7 (2)	C19—C10—C11—C12	30.49 (17)
C4—N1—C1—C2	172.65 (11)	N3—C10—C19—C14	177.77 (13)
C1—N1—C3—S1	−4.18 (14)	N3—C10—C19—C18	−0.49 (19)
C1—N1—C3—N2	174.63 (12)	C11—C10—C19—C14	−1.97 (19)
C4—N1—C3—S1	−172.16 (9)	C11—C10—C19—C18	179.77 (13)
C4—N1—C3—N2	6.65 (19)	C10—C11—C12—C13	−56.61 (16)
C1—N1—C4—C5	−79.47 (16)	C11—C12—C13—C14	54.64 (17)
C1—N1—C4—C9	101.58 (15)	C12—C13—C14—C15	152.42 (15)
C3—N1—C4—C5	87.64 (16)	C12—C13—C14—C19	−26.8 (2)
C3—N1—C4—C9	−91.31 (15)	C13—C14—C15—C16	−178.97 (16)
C3—N2—N3—C10	179.44 (11)	C19—C14—C15—C16	0.2 (2)
N3—N2—C3—S1	4.12 (16)	C13—C14—C19—C10	0.0 (2)
N3—N2—C3—N1	−174.53 (11)	C13—C14—C19—C18	178.25 (14)
N2—N3—C10—C11	5.64 (19)	C15—C14—C19—C10	−179.24 (13)
N2—N3—C10—C19	−174.09 (11)	C15—C14—C19—C18	−1.0 (2)
O1—C1—C2—S1	175.85 (12)	C14—C15—C16—C17	0.3 (3)
O1—C1—C2—C20	−2.9 (2)	C15—C16—C17—C18	−0.2 (3)
N1—C1—C2—S1	−3.45 (14)	C16—C17—C18—C19	−0.6 (3)
N1—C1—C2—C20	177.83 (12)	C17—C18—C19—C10	179.44 (14)
S1—C2—C20—C21	−2.6 (2)	C17—C18—C19—C14	1.2 (2)
C1—C2—C20—C21	175.97 (12)	C2—C20—C21—O2	−4.3 (2)
N1—C4—C5—C6	−178.63 (12)	C2—C20—C21—O3	176.24 (12)

### Hydrogen-bond geometry (Å, º)

**Table:**

*D*—H···*A*	*D*—H	H···*A*	*D*···*A*	*D*—H···*A*
C5—H5···O2^i^	0.95	2.59	3.3704 (18)	140
C11—H11*B*···N2	0.99	2.38	2.7466 (18)	101
C20—H20···O3^ii^	0.95	2.56	3.4591 (16)	159
C22—H22*C*···O1^ii^	0.98	2.52	3.4397 (18)	157

Symmetry codes: (i) −*x*+1, −*y*+2, −*z*+1; (ii) −*x*+2, −*y*+2, −*z*+1.

## References

[bb1] Aneja, D. K., Lohan, P., Arora, S., Sharma, C., Aneja, K. R. & Prakash, O. (2011). *Org. Med. Chem. Lett.* **1**, 1–11. 10.1186/2191-2858-1-15 PMC3320062 22373217

[bb2] Bruker (2013). *APEX2*, *SAINT* and *SADABS* Bruker AXS Inc., Madison, Wisconsin, USA.

[bb3] Cremer, D. & Pople, J. A. (1975). *J. Am. Chem. Soc.* **97**, 1354–1358.

[bb4] Desmet, C., Warze’e, B., Gosset, P., Me’lotte, D., Rongvaux, A., Gillet, L., Fie’vez, L., Seumois, G., Vanderplasschen, A., Staels, B., Lekeux, P. & Bureau, F. (2005). *Biochem. Pharmacol.* **69**, 255–265. 10.1016/j.bcp.2004.09.017 15627478

[bb5] Edelman, S. V. (2003). *Rev. Cardiovasc. Med.* **4**, S29–S37. 14668701

[bb6] Farrugia, L. J. (2012). *J. Appl. Cryst.* **45**, 849–854.

[bb7] Gillies, P. S. & Dunn, C. J. (2000). *Drugs*, **60**, 333–343. 10.2165/00003495-200060020-00009 10983737

[bb8] Lenhard, M. J. & Funk, W. B. (2001). *Diabetes Care*, **24**, 168–169. 10.2337/diacare.24.1.168 11194222

[bb9] Mague, J. T., Akkurt, M., Mohamed, S. K., Hassan, A. A. & Albayati, M. R. (2014). *Acta Cryst.* E**70**, o366–o367. 10.1107/S1600536814004048 PMC3998438 24765050

[bb10] Patel, D., Kumari, P. & Patel, N. (2010). *Arch. Appl. Sci. Res.* **2**, 68–75.

[bb11] Sheldrick, G. M. (2008). *Acta Cryst.* A**64**, 112–122. 10.1107/S0108767307043930 18156677

[bb12] Spek, A. L. (2009). *Acta Cryst.* D**65**, 148–155. 10.1107/S090744490804362X PMC2631630 19171970

